# Titania-Based Oxide Catalysts for Removing Nitrogen Oxides

**DOI:** 10.3390/ma19010020

**Published:** 2025-12-20

**Authors:** Anna Białas, Natalia Kowalska, Małgorzata Zimowska, Grzegorz Mordarski, Jacek Gurgul

**Affiliations:** 1 AGH University of Krakow, Faculty of Energy and Fuels, Mickiewicza 30, 30-059 Krakow, Poland; 2 Jerzy Haber Institute of Catalysis and Surface Chemistry, Polish Academy of Sciences, Niezapominajek 8, 30-239 Krakow, Poland; malgorzata.zimowska@ikifp.edu.pl (M.Z.); nbmordar@cyf-kr.edu.pl (G.M.); jacek.gurgul@ikifp.edu.pl (J.G.)

**Keywords:** sol–gel, titania, cerium, iron, SCR NO

## Abstract

Titania catalysts containing cerium, copper, or iron were obtained using the sol–gel method and tested in the selective reduction of nitrogen oxide. Samples with cerium and iron showed high activity at temperatures ranging from 200 to 400 °C, without the formation of N_2_O. The materials crystallized in anatase structure, and only a small amount of ceria was detected by XRD. Their crystallites were nanometric in size. The solids were mesoporous, with a specific surface area between 74 and 160 m^2^/g, determined based on nitrogen sorption at low temperature. The optimum Ce/Ti and Fe/Ti atomic ratio was 0.1 to 0.9, and such catalysts were composed of small anatase crystallites, although the presence of ceria also resulted in high catalytic activity. This activity was due to the presence of Fe^3+^ or Ce^3+^ ions on the surface of the material.

## 1. Introduction

Nitrogen oxides, produced during the combustion of fossil fuels, are one of the main factors contributing to air pollution. The most efficient way to remove them is selective catalytic reduction (SCR) using ammonia. Among commercially available catalysts, transition metal oxides are the active phase [1]. Metal dispersion and interaction between components are key factors influencing catalytic activity. Titanium oxide is applied as a dispersive phase because it interacts weakly with SO_2_ and is therefore resistant to poisoning by sulfur compounds. The adsorption of SO_2_ does not occur on mesoporous TiO_2_, and its layer can protect Fe_2_O_3_ and CeO_2_ catalysts. Titania doped with appropriate metals exhibits strong Lewis acidity, which is responsible for low-temperature activity in the SCR process. The addition of cerium increases the reactivity of such systems in the SCR NO_x_ process with ammonia due to the presence of Ti–O–Ce sites, which are also characterized by high hydrothermal stability. Ti–O–Ce atomic interactions cause an expansion of the SCR temperature range and surface development [1,2]. In such systems, a high Ce^3+^/Ce^4+^ ratio is important because it influences the presence of a greater amount of chemisorbed and labile oxygen [3]. Ce^3+^ ions cause strong Brønsted acidity, resulting in high-temperature SCR activity. CeO_2_ exhibits good redox properties and oxygen storage capacity due to oxygen vacancies [4]. Nanocrystalline CeO_2_ obtained by the sol–gel method provides better NH_3_ adsorption, redox capabilities, and catalytic activity. Fe_2_O_3_ is being tested as an additive to titania due to its environmental friendliness and thermal stability. This oxide exhibits medium- and high-temperature activity in the SCR process, is selective towards nitrogen, and is resistant to SO_2_. On the other hand, its narrow temperature window and reactivity at low temperatures are unsatisfactory. However, its deposition on the TiO_2_ (001) plane enables rapid electron transfer to Fe^3+^, regenerating this site, which is important in SCR. Fe^3+^ should be in a tetrahedral position, enhancing catalytic activity. Ti^4+^ doping of Fe_2_O_3_ increases acidity, as well as the number of oxygen defects and active sites [3]. In copper containing catalysts, its dispersion is most important—isolated and dimeric sites are catalytically active. Cu-Ti-O systems from coprecipitated precursors exhibit good acid and redox properties and high copper dispersion. They were active at 200 °C but operate in a narrow temperature range [3].

For the investigated Cu, Ce, and Fe/TiO_2_ systems, various metal ratios were described in the literature [4,5,6]. Ceria–titania catalytic supports exhibited the best performance (80% NO conversion, T = 200–300 °C) for the molar Ce/Ti ratio equal to 4:10 [5]. In this sample, with S_BET_ about 91 m^2^/g, ceria crystallites were detected. Cerium, iron, and copper, among other metals, were supported on titania nanotubes in the amount of 15wt.% [6], which corresponds to the molar ratio of Ce_0_,_1_Ti_0,9_ in the case of the cerium-containing sample. Over these catalysts, nitrogen oxides were removed totally in the temperature range of 150–300 °C. Optimization of the Ce, Fe, and Ti ratio in oxide systems was performed as well [4]. Results of this investigation revealed that the mixed oxide system, which contained Fe, Ce, and Ti with the molar ratio of 1:2:10, respectively, was the best catalyst. Only slightly worse was the sample with the ratio 1:1:10. Such iron content ensured only the presence of Fe_2_O_3_, with Fe^+3^ ions being important in the catalytic reaction. The lower Ce concentrations brought about its good reducibility and the presence of Ce^+3^ ions_,_ oxygen vacancies, and chemisorbed oxygen. We prepared the samples with similar metal concentrations, although we omitted the ternary metal samples in this manuscript because in our investigation, they resulted in lower catalytic activity than samples which contained only two metals. We decided to choose the molar Fe(Ce, Cu)/Ti ratio equal to 1:9 because such a metal concentration in TiAl mixed oxide resulted in its best performance as the support of a copper catalyst for SCR of NO with NH_3_ in our previous work [7].

The aim of this work is to develop a method for obtaining oxide systems in which titanium oxide is the dispersion phase and the addition of metal ensures high catalytic activity in the SCR NO process with ammonia without the formation of nitrous oxide. The sol–gel method was selected for the synthesis of nanocrystalline particles.

## 2. Materials and Methods

### 2.1. Synthesis

Glacial acetic acid (J.T. Baker, Deventer, The Netherlands) was added to titanium isopropoxide (Acros Organics, Geel, Belgium) in an atomic ratio of 10:1 and stirred for 30 min on a magnetic stirrer. An aqueous solution of metal nitrate (Ce—Acros Organics, Geel, Belgium, Cu and Fe—CHEMPUR, Piekary Śląskie, Poland) was added dropwise to this mixture. Prior to this, the appropriate salt was dissolved in water mixed with acetic acid in a 10:1 molar ratio.

The resulting solution was placed in a water bath at 80 °C and stirred until complete evaporation (3 to 5 h). The precipitate was then dried overnight at 80 °C. The ground powder was calcined at 450 °C for 3 h, the temperature being reached at a rate of 10 °C/min. One series of samples was prepared with a molar ratio of Ti to Ce, Cu, or Fe of 9:1 and designated as Ce_0.10_Ti_0.90_, Cu_0.10_Ti_0.90_, and Fe_0.10_Ti_0.90_. In the second series, this ratio was 9.5:0.5, yielding the samples designated as Ce_0.05_Ti_0.95_ and Fe_0.05_Ti_0.95_. In the third series, the ratio was 8.5:1.5, producing samples with the compositions Ce_0.15_Ti_0.85_ and Fe_0.15_Ti_0.95._

### 2.2. Catalytic Activity

The obtained samples were subjected to catalytic testing in SCR of NO with NH_3_. The powder samples were compressed and sieved. An amount of 0.2 g of the 0.25–1 mm fraction was placed in a tubular quartz microreactor on quartz wool. The catalytic bed was degassed at 450 °C for 1 h in helium and then cooled. The test was started at 150 °C and carried out at up to 450 °C in 50 °C increments, with each temperature maintained for 30 min. The concentration of NO and NH_3_ was 800 ppm, the O_2_ content was 3.5%, and these reagents were passed through a helium stream at a flow rate of 100 cm^3^/min. The stability test was carried out under the same conditions at 250 °C for 22 h. ABB IR AO2020 analyzers (Frankfurt am Main, Germany) were used to determine the concentration of NO and N_2_O in the products. NO conversion was calculated using the following formula:

$$K=\frac{C_{NOin}-C_{NOout}}{C_{NOin}}\times 100\%$$

where *C_NOin_* and *C_NOout_* indicates the concentration of *NO* at the inlet and outlet, respectively.

### 2.3. Composition, Structural and Textural Properties

Metal ratios in samples were determined with XR fluorescence by means of an EDX 3600 H apparatus manufactured by Skyray Instrument (Dallas, TX, USA), with a tungsten lamp (voltage 40 kV, current 450 μA). The time of a measurement was 100 s. Calibration curves were prepared for the commercial oxide samples mixed with the metal ratios corresponding to these intended in the syntheses. Powder X-ray diffraction (XRD) patterns were recorded using a PANalytical-Empyrean diffractometer (Malvern, UK) with a copper tube emitting CuK_α_ radiation (1.5406 Å) in the 2*θ* range between 5 and 90°. The investigation of the morphology and elemental composition in micro-areas of the titania-doped samples was carried out with the use of a JEOL JSM–7500F Field Emission Scanning Electron Microscope (JEOL, Akishima, Japan) equipped with a retractable backscattered-electron detector (RBEI) and the Aztec Live Lite Xplore 30 (Oxford Instruments, London, UK) energy dispersive X-ray spectroscopy (EDS) detector with Aztec Live Oxford Instruments Nano Analysis software version 5.1 for EDS analysis. Low-temperature nitrogen sorption was conducted to determine the specific surface area using the BET method (SSA_BET_) and the pore volume using the single-point method, as well as the distribution using the BJH method. A Micromeritics 3FLEX sorptomate (Norcross, GA, USA) was used to record the isotherms.

### 2.4. Surface Composition

X-ray photoelectron spectroscopy (XPS) measurements were carried out using a hemispherical analyzer (SES R4000, Gammadata Scienta, Uppsala, Sweden), a non-monochromatized AlK_α_ X-ray source operating at 12 kV, and an emission current of 15 mA. More details on the experimental part of XPS can be found in other publications [7,8].

## 3. Results and Discussion

### 3.1. Catalytic Activity

**Figure 1:**
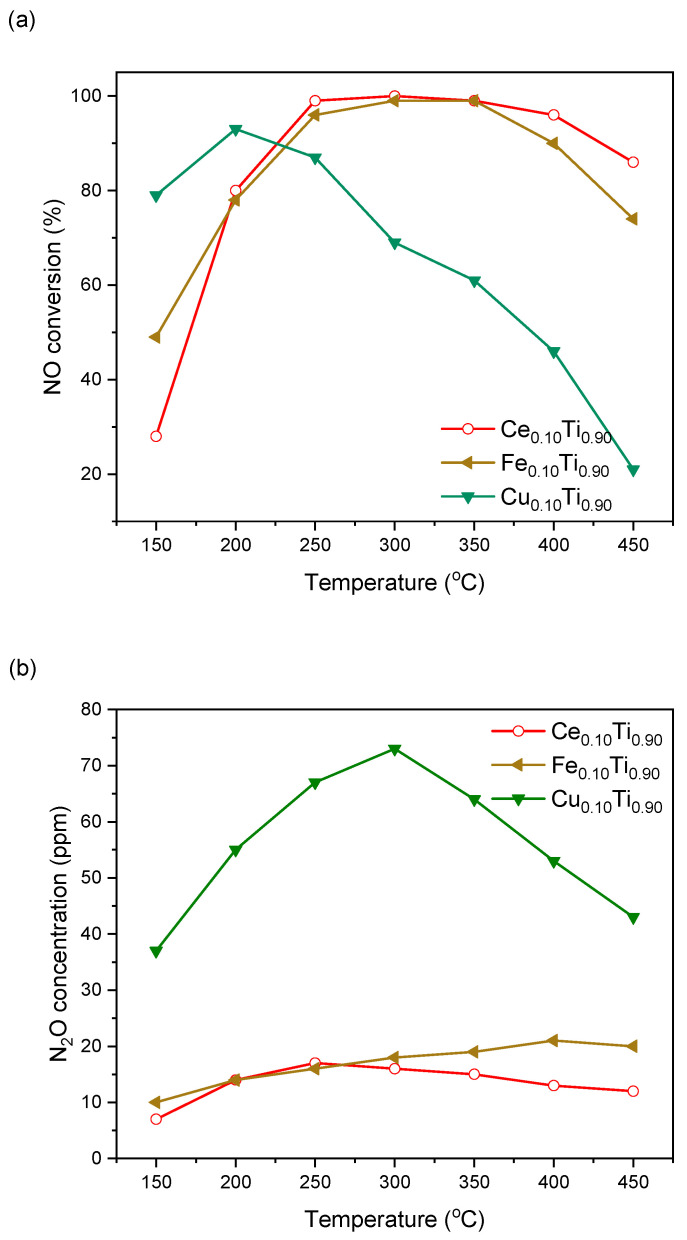
NO conversion (**a**) and N_2_O formation (**b**) over Ce(Cu, Fe)–titania catalysts.

The first series of samples with a molar ratio of added-metal-to-titanium of 1:9 was subjected to catalytic testing in SCR of NO with ammonia. As can be seen in Figure 1, the catalyst containing copper showed the highest activity at low temperatures. It achieved approximately 80% NO conversion at 150 °C and a maximum conversion of over 90% at 200 °C. Above this temperature, NO conversion dropped to 20% at 450 °C. In the case of the cerium and iron-containing catalysts, their activity at lower temperatures was not as high—close to 80% at 200 °C, but at 250 °C they exhibited NO conversion of approximately 100% and maintained it up to 350 °C. The Ce_0.1_Ti_0.9_ sample was highly active up to a temperature of 400 °C. During NO reduction, the formation of N_2_O was observed. In the case of the Cu_0.1_Ti_0.9_ sample, the concentration of N_2_O increased with temperature to over 70 ppm at 300 °C, and at the temperature of its highest activity it was around 60 ppm. For samples containing cerium and iron, the formation of this greenhouse gas was well below 30 ppm across the entire temperature range. 

**Figure 2:**
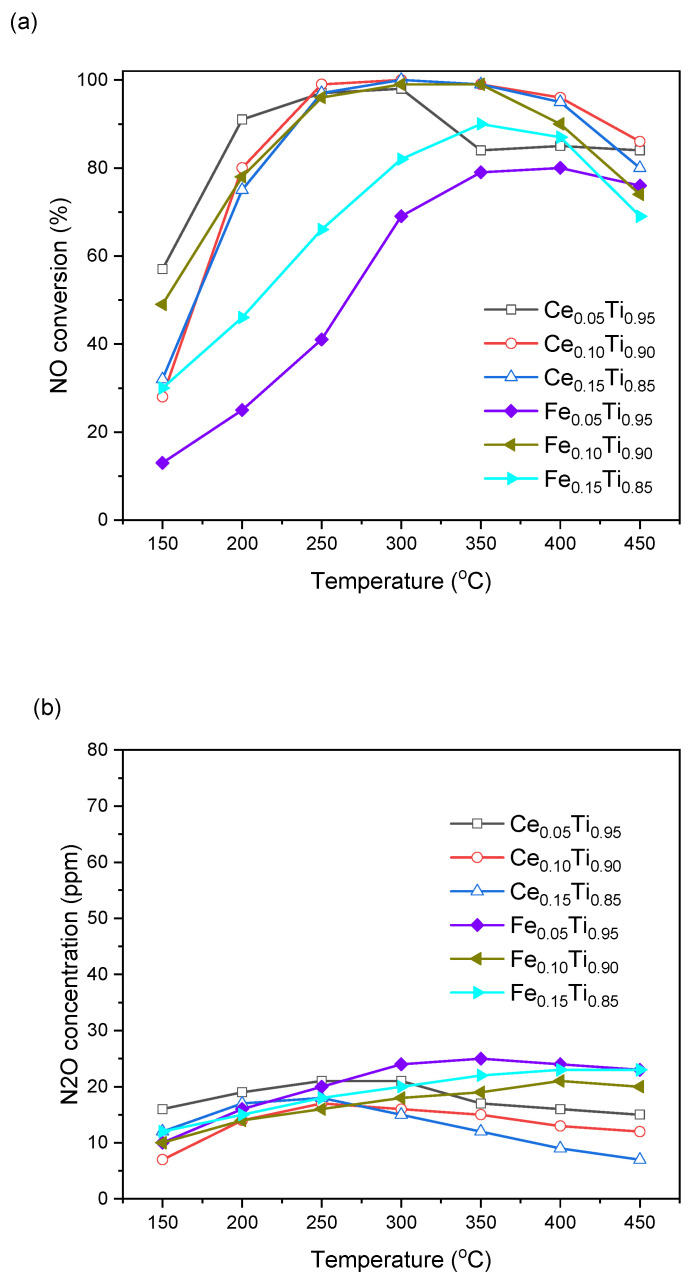
NO conversion (**a**) and N_2_O formation (**b**) over Ce and Fe titania catalysts.

Since samples containing cerium and iron exhibited high activity in a wider temperature range and negligible nitrous oxide formation, further catalysts with lower and higher concentration of these metals were prepared. As can be seen in Figure 2, the change in iron content caused a decrease in NO conversion, although N_2_O formation remained low. Such low concentrations were also observed for catalysts with different cerium contents. The effect of cerium concentration was less pronounced, with the Ce_0.15_Ti_0.85_ sample showing similar activity to the Ce_0.10_Ti_0.90_ catalyst and the sample with a lower Ce content showing only slightly lower activity.

The most active catalysts obtained in our syntheses exhibited their best performance (>90% NO conversion) in a broad temperature range (250–400 °C), which is comparable with the activity of samples reported in the literature (Table 1). The Ce_0.1_Ti_0.9_ sample was subjected to a stability test carried out at 250 °C for 22 h. No changes in NO conversion and N_2_O formation were detected (Figure S1), confirming thermal stability of the catalyst under reaction conditions.

### 3.2. Composition, Structural and Textural Properties

**Figure 3:**
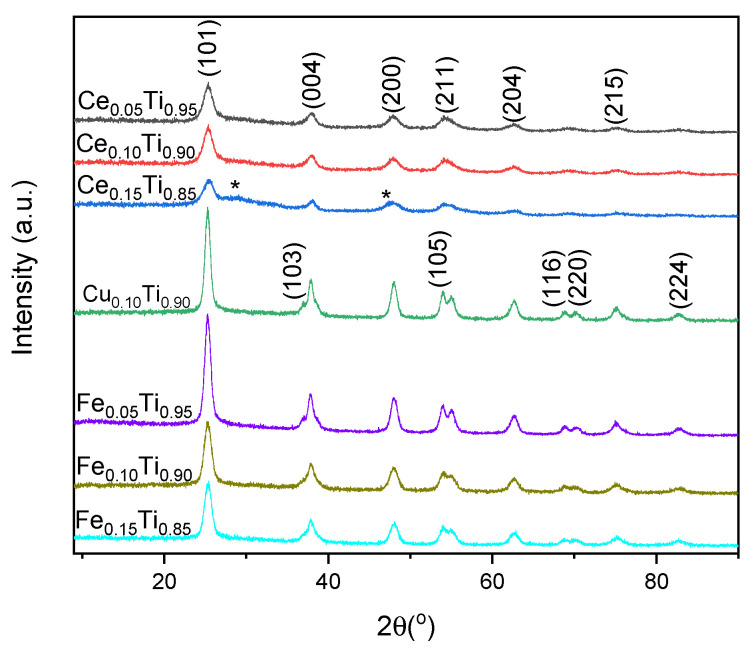
XRD patterns of Ce(Cu, Fe) titania samples, with (hkl) indexes of anatase; maxima marked with * originate from ceria.

To explain the catalytic activity of the samples, their composition, structural and textural, as well as surface, properties were determined. For all samples, the ratio of metals to titanium was slightly higher than calculated before syntheses (Table 2). In the case of the M_0.05_T_0.95_ catalysts, this surplus was about 5%; for M_0.10_T_0.90_ preparations, it was 10%, and in M_0.15_T_0.85_ samples, about 18% excess was detected. All of the oxide systems studied crystallized in the anatase structure. Maxima in the XRD patterns (Figure 3) registered at 2θ ca. 25.4, 36.9, 37.9, 48.0, 54.1, 55.1, 62.6, 68.9, 70.1, 75.2, and 82.9° can be ascribed to the (101), (103), (004), (200), (105), (211), (204), (116), (220), (215), and (224) planes of TiO_2_ anatase [01-071-1167], respectively. Only in the case of the Ce_0.15_Ti_0.85_ sample, two shoulders at ca. 28.7° and 47.3° originate from the (111) and (220) planes of CeO_2_, respectively [03-065-2975]. The added metals in the samples were incorporated into the titania structure or formed an amorphous phase. However, these additives affected the size of the crystallites. In general, a trend of decreasing crystallinity of the anatase structure with increasing amounts of added metal was observed. Cerium produced the smallest crystallites, while the sample with copper consisted of the largest crystallites. Their size was estimated using the Scherrer equation and was less than 11 nm for all samples (Table 2). In the case of samples containing cerium, an increase in Ce concentration resulted in a decrease in crystallite size to approximately 4 nm for the Ce_0.15_Ti_0.85_ sample, for which the appearance of a ceria phase was observed. This may confirm that, in small amounts, cerium oxide covers the surface of titanium oxide crystallites as an amorphous phase.

The analysis of the elemental composition (at.%) obtained on the basis of EDS measurements (Table 2) confirmed that the proportion of individual elements in the TiO_2_ structure is close to the intended one. However, it was observed that with an increase in nominal content (doping level), the amount of metal effectively introduced into the structure was decreased, which may indicate the formation of its oxide phase. This phenomenon is also reflected in the SE images of metal-doped TiO_2_ catalysts (Figure S2), which show an increase in the density of the introduced metal on the surface of the samples. In addition, at higher metal contents, small spots are observed, which may indicate the formation of extra-network metal oxides.

**Figure 4:**
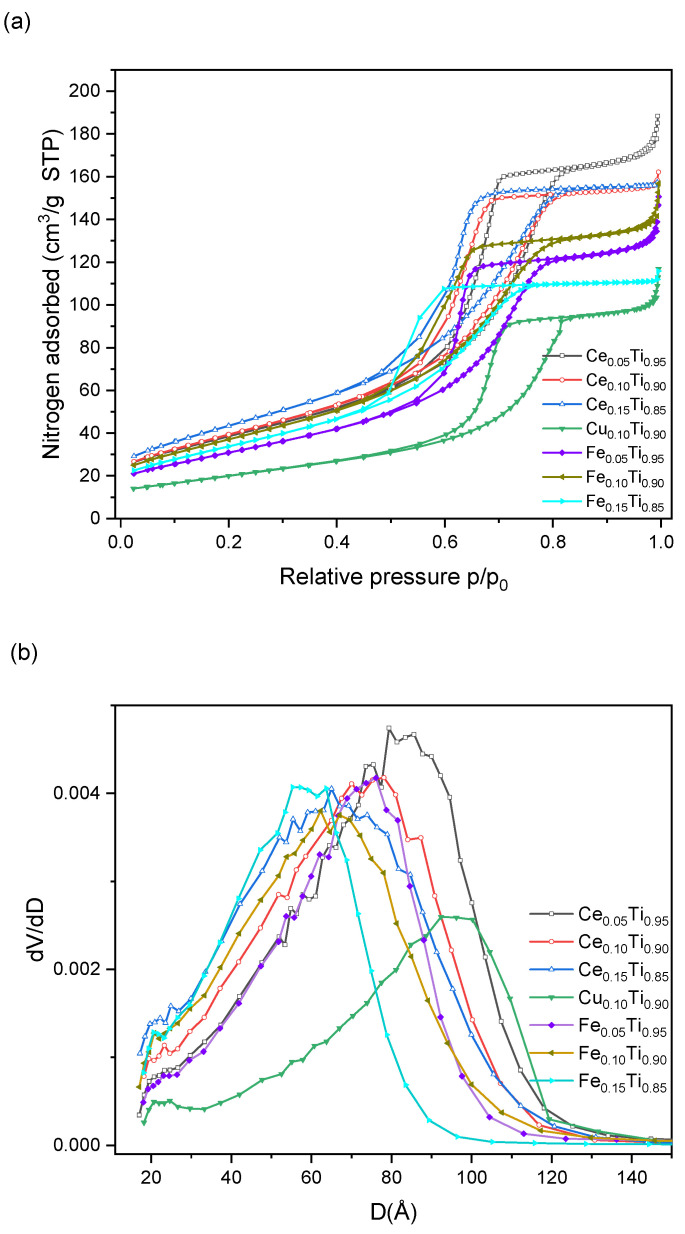
Nitrogen sorption isotherms (**a**) and volume pore distribution (**b**) for Ce(Cu, Fe) titania samples.

As can be seen in Figure 4a, the obtained preparations were mesoporous solids, which is confirmed by the shape of their nitrogen sorption isotherms. They can be classified as type IV according to the IUPAC classification [9]. The hysteresis loop can be classified as H2, typical for solids with irregular pore shapes and size distribution. The shape of the desorption branches suggests that the pores had necks of similar sizes and cavities of various diameters [10]. These conclusions are confirmed by the volume pore distribution (Figure 4b)—pores with sizes ranging from 60 to 100 Å dominated in the samples, and their distribution was wide. The widest pores were detected in the Cu_0.10_Ti_0.90_ catalyst, while the smallest ones were found in the Fe_0.15_Ti_0.85_ material.

As the concentration of the additional metal increased, the porosity decreased. The admixture of cerium to titania resulted in the highest SSA_BET_ and pore volume (Table 2), while these values were lower for oxides containing iron. Copper catalysts had the lowest surface area and pore volume. Cerium and iron catalysts exhibited SSA_BET_ values in the range of 114–160 m^2^/g, while the copper catalysts had almost half this surface area. The same relationship is observed for pore volume—preparations containing iron and cerium had a larger pore volume (0.174–0.275 cm^3^/g) than the copper catalyst (0.160 cm^3^/g) (Table 2).

Under working conditions, SCR catalysts can be poisoned, among others, by SO_2_ and water. The broad temperature window in which the obtained Fe and Ce/TiO_2_ samples are active enables their application above 300 °C, ensuring sulphates’ decomposition. It is also promoted by large mesopores (above 100 Å) [3] detected in the materials. But further investigation must be undertaken to confirm that these catalysts are proof of poisoning.

### 3.3. Surface Composition

To study the effect of dopants on the catalytic activity of TiO_2_ doped with cerium or iron, XPS studies of several samples with different Ce and Fe contents were performed before and after selective catalytic reduction of NO. The surface concentration of cerium and iron (in at.%) was calculated from XPS survey spectra (Table 3). The analyzed signal comes from a maximum depth of 8.5 nm, which was calculated according to the algorithm provided by Tanuma et al. [11], assuming that the samples are pure and homogeneous TiO_2_ with a density of 4.26 g cm^−3^. The calculations present 95% of all photoelectrons escaping from the surface. In the case of samples containing iron, the amount of this metal on the surface was slightly lower than the nominal concentration in the case of Fe_0.05_Ti_0.95_ and Fe_0.15_Ti_0.85_ catalysts. The surface of the Fe_0.10_Ti_0.90_ catalyst was enriched with iron by more than 30% compared to the intended amount and was almost equal to that in the Fe_0.15_Ti_0.85_ catalyst. SCR conditions caused an approximately 5% decrease in Fe content on the surface of the catalysts. For samples with cerium, a similar content to the nominal value was observed for the catalyst with lower metal concentrations, while in the catalyst with the highest Ce concentration, 50% excess of this metal was detected on its surface. The cerium content on the surface remained unchanged during the SCR reaction in the Ce_0.10_Ti_0.90_ catalyst. Such a situation did not occur in the case of the Ce_0.05_Ti_0.95_ sample—an approximately 20% increase in Ce content on the surface was observed, and in the case of the Ce_0.15_Ti_0.85_ catalyst, an approximately 20% decrease in this concentration was observed.

**Figure 5:**
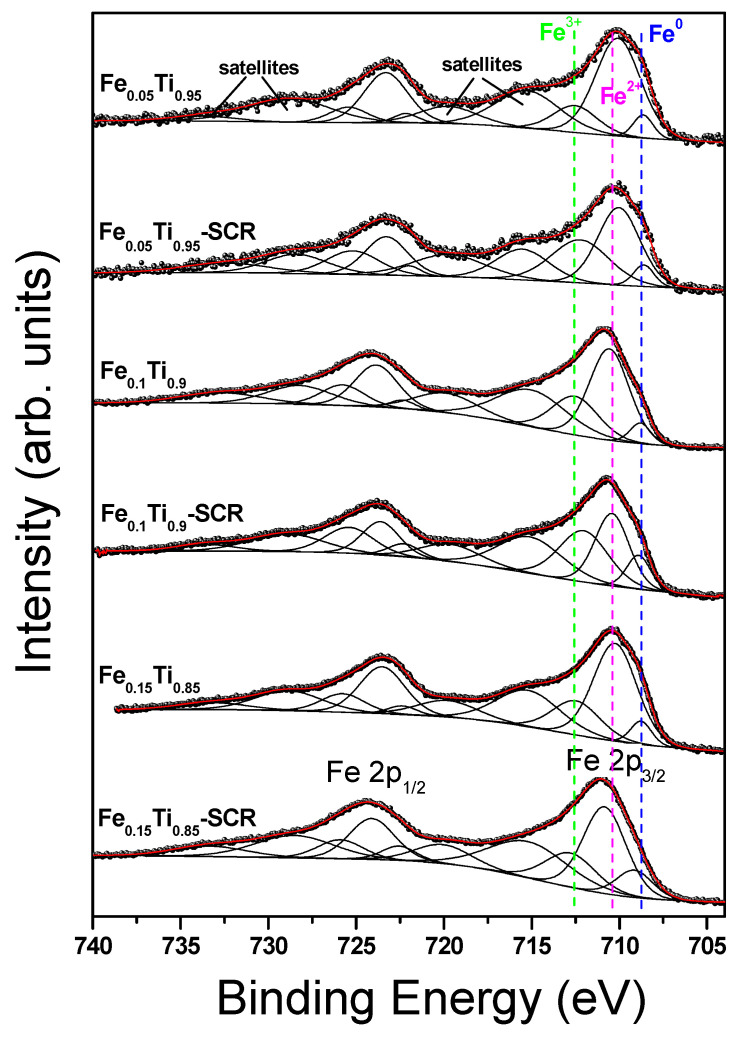
Fe 2p XPS spectra of titania doped with iron for fresh and used catalysts.

**Figure 6:**
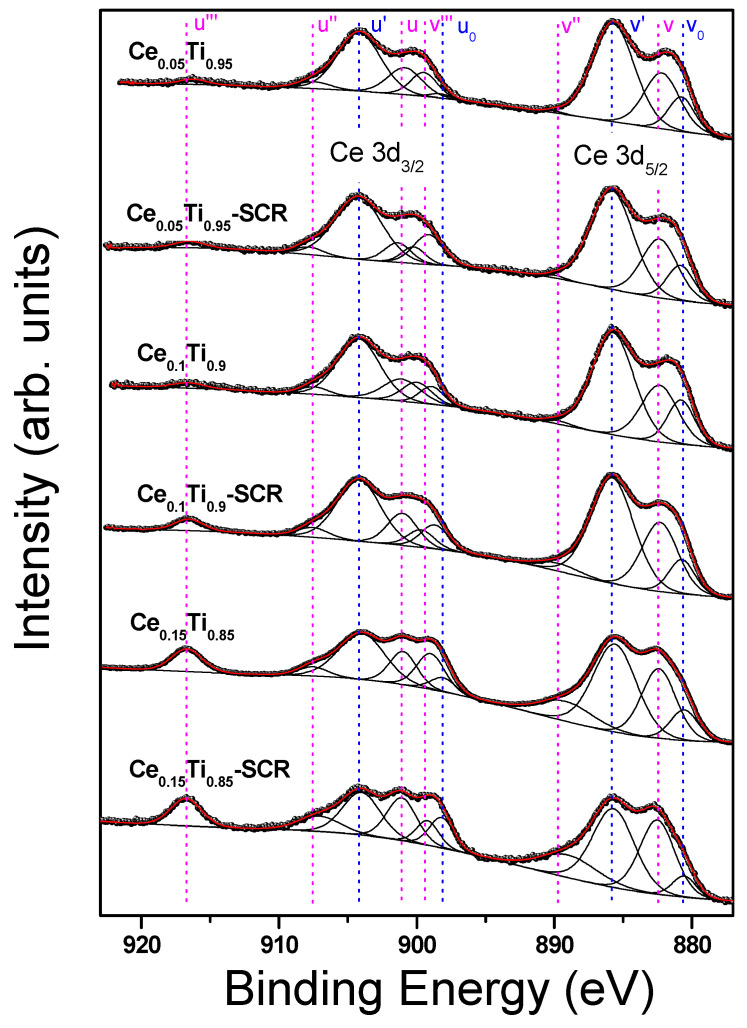
Ce 3d XPS spectra of titania doped with cerium for fresh and used catalysts. Ce^4+^ components are highlighted in pink, while Ce^3+^ components are highlighted in blue.

A detailed analysis of high-resolution XPS spectra was performed to obtain information on the oxidation states of the cations introduced into TiO_2_ and their changes following the SCR reaction. The results are shown in Figure 5 (Fe 2p) and Figure 6 (Ce 3d) and in the Supplementary Materials in Tables S1 (Ti 2p), S2 (C 1s) and S3 (O 1s).

The Ti 2p spectra of all samples were fitted perfectly with a single doublet of Ti 2p_3/2_ binding energy close to 458.7 eV (Table S1). Despite the fact that the spectra contain only a single component assigned to lattice Ti^4+^ ions in the anatase structure [12,13,14], the small contribution of M–O–Ti bonds cannot be completely ruled out, as they have little effect on the shape of the Ti 2p spectrum and are hardly noticeable due to the small shifts in binding energy [15]. Carrying out the SCR reaction does not cause the appearance of an additional component nor does it change the position of the Ti 2p line, which indicates the absence of changes in the TiO_2_ framework.

Three peaks at 285.0 eV (organic contaminants), 286.0–286.5 eV (C–O groups), and 288.8–289.1 eV (O–C=O groups) can be distinguished in the C 1s spectra (Table S2). Changes in the amount of C–O and carboxyl groups are negligible before and after the SCR reaction. The hydrocarbon contamination was used as an internal calibration for all samples, as was mentioned above.

The O 1s spectra were well decomposed into three components (Table S3): (i) the most intense peak (>80%) located at 529.9 eV relating to O^2−^ ions in the TiO_2_ lattice [12]; (ii) a small peak (<5%) at 528.1 eV associated with metal–O–Ti bonds [14]; (iii) a peak at BE of 531.6 eV assigned to OH groups and oxygen from organic contaminants (C–O, O–C=O functional groups) [12,13]. The second and most interesting component requires comment. It is known that Fe^3+^ ions with a radius of 0.64 Å can be incorporated into the TiO_2_ structure because Ti^4+^ ions have a slightly larger radius of 0.68 Å [16]. It is therefore likely that Fe^3+^ ions, whose presence has been confirmed by Fe 2p spectra, can form Fe–O–Ti bonds inside the TiO_2_ network. The formation of Fe–O–Ti bonds will increase the ionicity of the Ti–O bond, leading to a shift in the electron states of oxygen. Consequently, iron-doped TiO_2_ will have the O 1s line shifted toward lower binding energies compared to pure TiO_2_, which is in line with a paper by Nasser [17]. In contrast to the small ionic radius of Fe^3+^, both Ce^4+^ and Ce^3+^ have much larger ionic radii of 1.01 and 1.11 Å, respectively. For this reason, it is almost impossible for doped cerium ions to enter the TiO_2_ network and replace Ti^4+^ ions, forming stable solid solutions [16]. However, cerium ions can bind to the oxygen anion on the surface of TiO_2_ nanoparticles, and a Ce–O–Ti bond is formed at the CeO_x_-TiO_2_ interface. As shown earlier [15,18,19], at the interface, titanium ions sometimes replace cerium ions in the lattice of the surrounding cerium oxide. Table S3 shows that there are some slight differences in the relative amounts of oxygen species between fresh and used samples. However, it cannot be ruled out that these differences could have been larger, but samples after the SCR reaction were stored in an air atmosphere and may have reoxidized before XPS studies.

The Fe 2p spectra are quite complicated due to strong multiplet splitting and shake-up satellite phenomena (Figure 5). However, careful numerical analysis revealed the presence of three components: metallic, ferrous and ferric, and the accompanying two broad satellites. The presence of strong shake-up core-level satellites is characteristic of high-spin iron states, whereas low-spin states have weak or zero satellite structure [20]. It is worth recalling here that the pure metals do not show strong satellite structure in the 2p shell other than the usual plasmon losses [21].

The dominant contribution to the Fe 2p spectra (except Fe_0.10_Ti_0.90_-SCR sample) is the ferrous component (Table 4). After SCR reaction, a metallic component increases significantly in high-iron loaded catalysts (Fe_0.10_Ti_0.90_-SCR and Fe_0.15_Ti_0.85_-SCR), whereas it remains the same in the Fe_0.05_Ti_0.95_-SCR sample. The presence of significantly higher amounts of Fe^3+^ at the expense of a reduction in the amount of Fe^2+^ is observed in Fe_0.10_Ti_0.90_-SCR and Fe_0.05_Ti_0.95_-SCR samples. The SCR reaction does not influence the BE values of all components, as well as the BE of all satellites. Thus, one can conclude that the only change is the different number of specific iron species on the surface of studied catalysts.

The Ce 3d XPS spectra of Ce-doped TiO_2_ catalysts as received and after SCR are presented in Figure 6. According to the common approach [22], Ce 3d spectra were fitted with ten multiplet components marked “v” for Ce 3d_5/2_ and “u” for Ce 3d_3/2_ contributions (Table S4). The multiplicity of these states comes from the hybridization between the Ce 4f levels and the O 2p states. The peaks at 882.2 eV (v) and 890.0 eV (v′′), as well as at 900.9 eV (u) and 907.5 eV (u′′), can be assigned to a mixing of Ce(3d^9^4f^2^)O(2p^4^) and Ce(3d^9^4f^1^)O(2p^5^) Ce(IV) final states, whereas peaks at 899.5 eV (v′′′) and 916.3 eV (u′′′) correspond to the Ce(3d^9^4f^0^)O(2p^6^) Ce(IV) final state. On the other hand, 880.7 (v_0_), 885.7 (v′), 898.3 (u_0_), and 904.2 eV (u′) contributions can be attributed to the Ce(3d^9^4f^2^)O(2p^5^) and the Ce(3d^9^4f^1^)O(2p^6^) of the Ce(III) final state [23]. Thus, it can be concluded that XPS analysis revealed the coexistence of Ce^3+^ and Ce^4+^ ions on the surface of all samples. Moreover, the binding energies of the aforementioned peaks agree quite well with those of the peaks derived from CeO_2_ and Ce_2_O_3_ presented in the work of Romeo et al. [24].

The position of the Ce^4+^ reference line (u′′′) was 916.3 eV, in perfect agreement with the data from the literature [22,24]. The area under this line, which is a marker for the amount of Ce^4+^, suggests that the SCR reaction slightly increases the amount of Ce^4+^ ions on the surface. This observation is also confirmed by the surface concentration of Ce^3+^ estimated from the Ce^3+^/(Ce^3+^ + Ce^4+^) peak areas ratio [Table S4]. It is worth mentioning that the predominant oxidation state of cerium present on the surface of the samples studied is +3.

According to the literature, both cycles—redox and acidic—are important in SCR of NO with ammonia [3,4,25]. Based on DFT calculations, Liu proposed the four-step mechanism for tungsta–ceria catalysts [25]. Zhan presented the mechanism for iron–ceria–titania systems [4]. Combining these findings with our investigation, we can assume that NO reduction over Ce-titania, Fe-titania, and Cu-titania catalysts can occur as follows:

Proposed NH_3_–SCR mechanism over lattice-doped M–TiO_2_ (M = Ce, Cu, Fe):

Step I. NH_3_ adsorption and activation (Brønsted and Lewis sites).

- Formation of surface NHx species at Lewis acidic metal centres and oxygen vacancies:

$${NH}_{3}\left(g\right)+M_{TiO_{2}}^{n+}↔{NH}_{3}-M^{n+}\;{NH}_{3}+{OH}_{surf}^{-}\to {NH}_{2}^{*}+H_{2}O$$

Step II. NO adsorption and metal-assisted activation.

- Ce^4+^/Ce^3+^, Cu^2+^/Cu^+^, Fe^3+^/Fe^2+^ redox couples:

$$NO\left(g\right)+M_{TiO_{2}}^{n+}↔NO^{*}-M^{n+}\;M^{n+}+NO\to M^{\left(n-1\right)+}+NO^{*}$$

Step III. NO dimerization at oxygen vacancies.

- Stabilization of dinitrosyl species at lattice oxygen vacancies:

$$2NO+O_{v}\to {\left(N_{2}O_{2}\right)}_{O_{v}}^{*}$$

Step IV. Surface reaction between NH_x_* and activated NO—rate-determining step.

Direct pathway:

$$NH_{2}^{*}+NO^{*}\to N_{2}+H_{2}O+M^{\left(n-1\right)+}$$

Dimer-mediated pathway—occurs at the M–O_v interfacial sites:

$$2NH_{2}+{\left(N_{2}O_{2}\right)}^{*}\to 2N_{2}+2H_{2}O$$

Step V. Catalyst reoxidation and vacancy healing.

- Restoration of lattice oxygen and acidic surface sites:

$$O_{2}+2M^{\left(n-1\right)+}+O_{v}\to 2M^{n+}+O_{lattice}^{2-}\;O_{lattice}^{2-}+H^{+}\to OH_{surf}^{-}$$

where:

- NH_x_ = NH_2_*, NH* surface species;
- O_v_ = oxygen vacancy;
- Redox cycle: M^n+^ ⇌ M^n−1+^.

**Figure 7:**
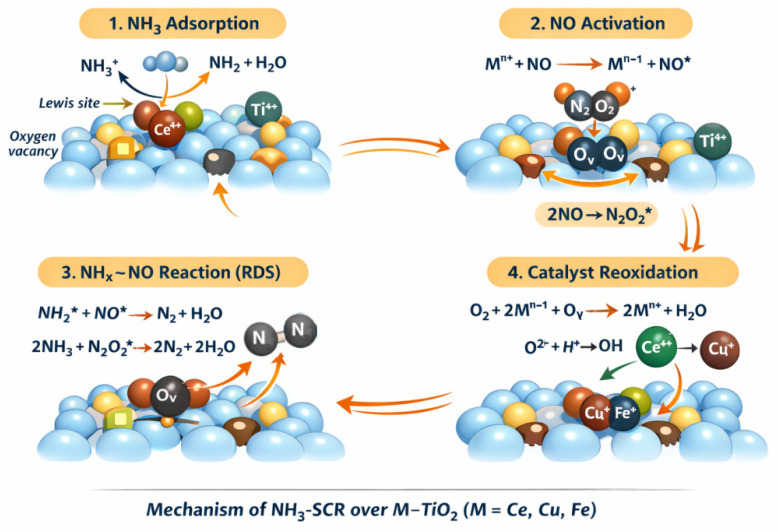
Proposed NH_3_–SCR mechanism over lattice-doped M–TiO_2_ (M = Ce, Cu, Fe).

Schematic representation of the NH_3_–SCR mechanism over TiO_2_ catalysts doped with redox-active metal cations (Ce, Cu, Fe) is presented in Figure 7. NH_3_ adsorption occurs on Brønsted acidic surface hydroxyls and Lewis acidic metal sites, generating NH_x_ surface species. NO is activated through interaction with reduced metal centers and oxygen vacancies, leading to the formation of NO-derived intermediates, including surface dinitrosyl species. The rate-determining step involves the surface reaction between NH_x_* and activated NO species at M–O_v_ interfacial sites, yielding N_2_ and H_2_O. Nitrogen formation proceeds via NH_x_–NO coupling at the metal–oxygen vacancy interface. Molecular oxygen serves as a reoxidant, restoring lattice oxygen, surface hydroxyl groups, and the oxidized state of the metal dopants. Catalyst reoxidation by O_2_ replenishes lattice oxygen, regenerates surface –OH groups, and restores the oxidized metal state, closing the catalytic cycle.

SCR catalysts should contain Lewis and Brønsted sites to be active in a broad temperature window like the Ce_0.10_Ti_0.90_ or Fe_0.10_Ti_0.90_ samples. In the case of the copper catalyst, we observed the lowest content of hydroxyls groups, and this sample was highly active only at low temperatures. In a similar system, we detected mainly Cu^+^ ions [7].

## 4. Conclusions

Mixed oxides of cerium (copper or iron) and titanium were obtained using the sol–gel method. The solids crystallized in the form of anatase, but cerium oxide was present in the sample with a Ce:Ti ratio of 0.15:0.85 as well. The size of the crystallites ranged from 4 to 11 nm. The samples were mesoporous solids with large pores, exhibiting a specific surface area above 100 m^2^/g for the cerium and iron–titania catalyst and approximately 75 m^2^/g for the copper catalyst. Cerium and iron–titanium oxides with a ratio 0.10:0.90, as well as the Ce_0.15_Ti_0.85_ sample, exhibited high catalytic activity (>70%) in SCR of NO without the formation of N_2_O in a wide temperature range (200–400 °C). This broad temperature window was caused by the presence of Lewis and Brønsted acid sites. In the case of both metals (Ce and Fe) added to titania, the concentration of +3 ions influenced their high catalytic activity. If Ce^+3^ ions were dominant, well dispersed, and stable under SCR conditions, they were responsible for activity at low temperatures (Ce_0.05_Ti_0.95_). Higher cerium concentrations caused instability in the Ce^+3^/Ce^+4^ ratio under reaction conditions but resulted in wider reaction temperature windows—approximately 100% conversion between 250 and 400 °C.

## Figures and Tables

**Table 1:** Comparison of catalytic activity, in SCR of NO with ammonia, for various Ce, Cu, Fe oxide systems.

Oxide Catalysts	Composition	Reaction Condition	Conversion	Ref.
Ce40/Ti100	Ce/Ti = 0.4	1000 ppm NO, 1000 ppm NH_3_, 5 vol% O_2_, He balance	~80% (T = 250–300 °C)	[5]
Ce(0.2)FeTi	Ce/Ti = 0.2 Fe/Ti = 0.1	750 ppm NO, 900 ppm NH_3_, 5 vol% O_2_, N_2_ balance	~100% (T = 225–300 °C)	[4]
Ce(0.1)FeTi	Ce/Ti = 0.1 Fe/Ti = 0.1	750 ppm NO, 900 ppm NH_3_, 5 vol % O_2_, N_2_ balance	~100% (T = 250–300 °C)	[4]
Ce/TNT	15%Ce/TiO_2_ nanotubes	900 ppm NO, 100 ppm NO_2_, 1000 ppm NH_3_, 10 vol% O_2_, He balance	100% (T = 200–300 °C)	[6]
Cu/TNT	15%Cu/TiO_2_ nanotubes	900 ppm NO, 100 ppm NO_2_, 1000 ppm NH_3_, 10 vol% O_2_, He balance	100% (T = 150–250 °C)	[6]
FeTi	Fe/Ti = 0.1	750 ppm NO, 900 ppm NH_3_, 5vol% O_2_, N_2_ balance	~50%(T = 300 °C)	[4]
Fe/TNT	15%Ce/TiO_2_ nanotubes	900 ppm NO, 100 ppm NO_2_, 1000 ppm NH_3_, 10 vol% O_2_, He balance	100% (T = 250–300 °C)	[6]

**Table 2:** Composition (at.%) obtained from XRF and EDS measurements, structural, and textural properties of Ce (Cu, Fe)–titania catalysts.

Sample	Measured Composition			Crystallite Size * [nm]	Specific Surface Area [m^2^/g]	Total Pore Volume [cm^3^/g]
	**XRF**	**EDS**				
		**M [at%]**	**Ti [at%]**			
Ce_0.05_Ti_0.95_	Ce_0.05_Ti_0.95_	4.8	95.2	7.5	142	0.275
Ce_0.10_Ti_0.90_	Ce_0.11_Ti_0.89_	8.5	91.5	6.5	145	0.244
Ce_0.15_Ti_0.85_	Ce_0.18_Ti_0.82_	14.0	86.0	4.2	160	0.242
Cu_0.10_Ti_0.90_	Cu_0.11_Ti_0.89_	10.8	89.2	10.6	74	0.160
Fe_0.05_Ti_0.95_	Fe_0.05_Ti_0.95_	2.0	98.0	8.9	114	0.208
Fe_0.10_Ti_0.90_	Fe_0.11_Ti_0.89_	7.7	92.3	7.4	137	0.219
Fe_0.15_Ti_0.85_	Fe_0.18_Ti_0.89_	9.8	90.2	7.6	126	0.174

* Calculated for (101) plane.

**Table 3:** Surface composition of iron- or cerium-doped titania (at.%) obtained from XPS survey scans.

Sample	Ti	Fe	Ce	O	C
Fe_0.05_Ti_0.95_	25.6	1.3	--	66.2	6.9
Fe_0.05_Ti_0.95_—SCR	25.4	1.2	--	65.8	7.6
Fe_0.10_Ti_0.90_	23.0	3.6	--	64.6	8.8
Fe_0.10_Ti_0.90_—SCR	22.1	3.3	--	62.9	11.7
Fe_0.15_Ti_0.85_	23.9	4.0	--	64.8	7.3
Fe_0.15_Ti_0.85_—SCR	23.8	3.7	--	65.4	7.1
Ce_0.05_Ti_0.95_	23.5	--	1.3	64.8	10.4
Ce_0.05_Ti_0.95_—SCR	25.3	--	1.7	65.8	7.2
Ce_0.10_Ti_0.90_	23.1	--	2.5	65.0	9.4
Ce_0.10_Ti_0.90_—SCR	23.1	--	2.4	64.7	9.8
Ce_0.15_Ti_0.85_	20.4	--	5.8	62.3	11.5
Ce_0.15_Ti_0.85_—SCR	20.7	--	5.0	64.3	10.0

**Table 4:** The binding energy of Fe 2p_3/2_ components, as well as shake-up satellites (eV) and relative areas (%, in parentheses) obtained for fresh and used catalysts.

Sample	Fe^0^	Fe^2+^	Fe^3+^	Sat	Sat	Sat	Sat
Fe_0.05_Ti_0.95_ Fe_0.05_Ti_0.95_-SCR Fe_0.10_Ti_0.90_ Fe_0.10_Ti_0.90_-SCR Fe_0.15_Ti_0.85_ Fe_0.15_Ti_0.85_-SCR	708.6 (7.5) 708.6 (7.2) 708.8 (7.3) 708.9 (14.0) 708.8 (7.7) 709.1	710.1 (73.0) 710.0 (52.7) 710.6 (62.2) 710.4 (42.7) 710.3 (66.8) 710.8	712.5 (19.5) 712.2 (40.1) 712.5 (30.5) 712.0 (43.3) 712.5 (25.5) 712.8	715.2 715.5 715.0 715.1 715.2 715.2	719.8 719.7 719.9 719.6 719.8 720.1	728.8 728.4 728.2 728.9 728.8 728.5	733.6 732.4 732.7 733.6 733.2 733.3
	(15.3)	(57.9)	(26.8)				

## Data Availability

The original contributions presented in this study are included in the article/Supplementary Material. Further inquiries can be directed to the corresponding author.

## Supplementary Materials

The following supporting information can be downloaded at: https://www.mdpi.com/article/10.3390/ma19010020/s1; Table S1: Numerical analysis of high-resolution XPS spectra of Ti 2p lines; Table S2: Numerical analysis of high-resolution XPS spectra of C1s lines. Table S3: Numerical analysis of high-resolution XPS spectra of O 1s lines. Table S4: Binding energy values (eV) of the Ce 3d lines dominated by multiplet effects. Figure S1: NO conversion (a) and N2O formation (b) over the Ce0.1Ti0.9 catalyst during stability test carried out at 250 °C for 22 h. Figure S2: SEM_EDS distribution maps of elements in Ce(Cu, Fe) titania samples.

## References

[1] Li J., Chang H., Ma L., Hao J., Yang R.T. (2011). Low-temperature selective catalytic reduction of NOx with NH_3_ over metal oxide and zeolite catalysts-A review. Catal. Today.

[2] Li P., Xin Y., Li Q., Wang Z., Zhang Z., Zheng L. (2012). Ce-Ti amorphous oxides for selective catalytic reduction of NO with NH_3_: Confirmation of Ce-O-Ti active sites. Environ. Sci. Technol..

[3] Han L., Cai S., Gao M., Hasegawa J.Y., Wang P., Zhang J., Shi L., Zhang D. (2019). Selective Catalytic Reduction of NO_x_ with NH_3_ by Using Novel Catalysts: State of the Art and Future Prospects. Chem. Rev..

[4] Zhan S., Zhu D., Yang S., Qiu M., Li Y., Yu H., Shen Z. (2015). Sol-gel preparation of mesoporous cerium-doped FeTi nanocatalysts and its SCR activity of NOx with NH_3_ at low temperature. J. Sol-Gel Sci. Technol..

[5] Mosrati J., Atia H., Eckelt R., Huyen T., Rabeah J., Mhamdi M., Armbruster U. (2021). Ta and Mo oxides supported on CeO_2_-TiO_2_ for the selective catalytic reduction of NO_x_ with NH_3_ at low temperature. J. Catal..

[6] Boningari T., Pappas D.K., Smirniotis P.G. (2018). Metal oxide-confined interweaved titania nanotubes M/TNT (M = Mn, Cu, Ce, Fe, V, Cr, and Co) for the selective catalytic reduction of NO_x_ in the presence of excess oxygen. J. Catal..

[7] Guziewicz W., Białas A., Napruszewska B.D., Zimowska M., Gurgul J. (2021). Aluminum doped titania as a support of copper catalysts for scr of nitrogen oxides. Materials.

[8] Białas A., Rugała K., Czosnek C., Mordarski G., Gurgul J. (2020). Copper aluminum spinels doped with cerium as catalysts for NO removal. Catalysts.

[9] Sing K.S.W., Everett D.H., Haul R.A.W., Moscou L., Pierotti R.A., Rouquerol J., Siemieniewska T. (1985). Reporting Physisorption Data for Gas/Solid Systems with Special Reference to the Determination of Surface Area and Porosity. Pure Appl. Chem..

[10] Cychosz K.A., Thommes M. (2018). Progress in the Physisorption Characterization of Nanoporous Gas Storage Materials. Engineering.

[11] Tanuma S., Powell C.J., Penn D.R. (1993). Calculations of electron inelastic mean free paths. V. Data for 14 organic compounds over the 50–2000 eV range. Surf. Interface Anal..

[12] Greczynski G., Hultman L. (2016). Self-consistent modelling of X-ray photoelectron spectra from air-exposed polycrystalline TiN thin films. Appl. Surf. Sci..

[13] Bertóti I., Mohai M., Sullivan J.L., Saied S.O. (1995). Surface characterisation of plasma-nitrided titanium: An XPS study. Appl. Surf. Sci..

[14] Barreca F., Acacia N., Barletta E., Spadaro D., Currò G., Neri F. (2010). Small size TiO_2_ nanoparticles prepared by laser ablation in water. Appl. Surf. Sci..

[15] Xu Y.H., Zeng Z.X. (2008). The preparation, characterization, and photocatalytic activities of Ce-TiO_2_/SiO_2_. J. Mol. Catal. A Chem..

[16] Hung W.C., Chen Y.C., Chu H., Tseng T.K. (2008). Synthesis and characterization of TiO_2_ and Fe/TiO_2_ nanoparticles and their performance for photocatalytic degradation of 1,2-dichloroethane. Appl. Surf. Sci..

[17] Nasser S.A. (2000). X-ray photoelectron spectroscopy study on the composition and structure of BaTiO_3_ thin films deposited on silicon. Appl. Surf. Sci..

[18] Lin J., Yu J.C. (1998). An investigation on photocatalytic activities of mixed TiO_2_-rare earth oxides for the oxidation of acetone in air. J. Photochem. Photobiol. A Chem..

[19] Liu Y., Wei J.H., Xiong R., Pan C.X., Shi J. (2011). Enhanced visible light photocatalytic properties of Fe-doped TiO_2_ nanorod clusters and monodispersed nanoparticles. Appl. Surf. Sci..

[20] Corneille J.S., He J.W., Goodman D.W. (1995). Preparation and characterization of ultra-thin iron oxide films on a Mo(100) surface. Surf. Sci..

[21] Stucky G.S., Carlson T.A., Vernon G.A. (1976). Comprehensive Study of Satellite Structure in the Photoelectron Spectra of Transition Metal Compounds. Inorg. Chem..

[22] Paparazzo E. (2018). Corrigendum: Use and mis-use of x-ray photoemission Ce3d spectra of Ce_2_O_3_ and CeO_2_. J. Phys. Condens. Matter.

[23] Yousefi M., Azimirad R., Amiri M., Moshfegh A.Z. (2011). Effect of annealing temperature on growth of Ce-ZnO nanocomposite thin films: X-ray photoelectron spectroscopy study. Thin Solid Films.

[24] Romeo M., Bak K., El Fallah J., Le Normand F., Hilaire L. (1993). XPS Study of the reduction of cerium dioxide. Surf. Interface Anal..

[25] Liu B., Liu J., Ma S., Zhao Z., Chen Y., Gong X., Song W. (2016). Mechanistic Study of Selective Catalytic Reduction of NO with NH_3_ on W—Doped CeO_2_ Catalysts: Unraveling the Catalytic Cycle and the Role of Oxygen Vacancy. J. Phys. Chem. C.

