# Supplementary material for: Titania-Based Oxide Catalysts for Removing Nitrogen Oxides
Source: Materials (Basel). 2025 Dec 20;19(1):20. doi: 10.3390/ma19010020 (PMC12786766; doi:10.3390/ma19010020)
Supplement: Supplementary file 1 [file materials-19-00020-s001.zip › materials-3985112-supplementary.pdf]

Titania-Based Oxide Catalysts for Removing Nitrogen Oxides

Anna Białas[1*], Natalia Kowalska[1], Małgorzata Zimowska[2], Grzegorz Mordarski[2], Jacek Gurgul[2]

[1]AGH University of Krakow, Faculty of Energy and Fuels, Mickiewicza 30, 30-059 Krakow, Poland

[2]Jerzy Haber Institute of Catalysis and Surface Chemistry, Polish Academy of Sciences, Niezapominajek 8, 30-239 Krakow, Poland

Data obtained by performing numerical analysis of high-resolution XPS spectra of Ti 2p, C 1s and O 1s lines.

Table S1. Data obtained by performing numerical analysis of high-resolution XPS spectra of Ti 2p lines. Since the spectra were fitted only with a single doublet, therefore, the table gives the binding energy values for Ti $2p_{3/2}$ lines only.

Sample	BE of Ti $2p_{3/2}$
$Ce_{0.05}Ti_{0.95}$	458.7
$Ce_{0.05}Ti_{0.95}$-SCR	458.7
$Ce_{0.10}Ti_{0.90}$	458.6
$Ce_{0.10}Ti_{0.90}$-SCR	458.7
$Ce_{0.15}Ti_{0.85}$	458.6
$Ce_{0.15}Ti_{0.85}$-SCR	458.5
$Fe_{0.05}Ti_{0.95}$	458.6
$Fe_{0.05}Ti_{0.95}$-SCR	458.6
$Fe_{0.10}Ti_{0.90}$	458.6
$Fe_{0.10}Ti_{0.90}$-SCR	458.6
$Fe_{0.15}Ti_{0.85}$	458.6
$Fe_{0.15}Ti_{0.85}$-SCR	458.7
$Cu_{0.10}Ti_{0.90}$	458.7
$Cu_{0.10}Ti_{0.90}$-SCR	458.7

Ti $2p_{3/2}$:$2p_{1/2}$ area ratio was constrained to 2:1. BE of TiO_2 is referenced to 458.7 eV [G. Greczynski, L. Hultman, Appl. Surf. Sci. 387 (2016) 294-300; I. Bertoti, M. Mohai, J. L. Sullivan, S. O. Saied, Appl. Surf. Sci. 84 (1995) 357-371; V. I. Bukhtiyarov, Catal. Today 56 (2000) 403-414; D. Jaeger, J. Patscheider, J. Electron. Spec. Rel. Phenom. 185 (2012) 523-534].

Table S2. Data obtained by performing numerical analysis of high-resolution XPS spectra of C1s lines. The BE values (eV) and relative areas of components (%) are given.

Sample	C-C/C-H	C-O	O-C=O
$Ce_{0.05}Ti_{0.95}$	285.0	286.4	288.9
	(61.3)	(16.6)	(22.1)
$Ce_{0.05}Ti_{0.95}$-SCR	285.0	286.4	288.9
	(64.2)	(11.4)	(24.4)
$Ce_{0.10}Ti_{0.90}$	285.0	286.3	289.1
	(54.4)	(17.6)	(28.0)
$Ce_{0.10}Ti_{0.90}$-SCR	285.0	286.0	289.0
	(45.4)	(28.3)	(26.3)
$Ce_{0.15}Ti_{0.85}$#	285.0	286.2	289.0
	(49.8)	(20.0)	(28.0)
$Ce_{0.15}Ti_{0.85}$-SCR	285.0	286.5	288.9
	(61.6)	(10.8)	(27.6)
$Fe_{0.05}Ti_{0.95}$	285.0	286.2	289.0
	(60.0)	(16.9)	(23.1)
$Fe_{0.05}Ti_{0.95}$-SCR	285.0	286.4	288.8
	(59.7)	(15.2)	(25.1)
$Fe_{0.10}Ti_{0.90}$	285.0	286.1	289.0
	(47.8)	(30.4)	(21.8)
$Fe_{0.10}Ti_{0.90}$-SCR	285.0	286.1	289.1
	(47.4)	(34.5)	(18.1)
$Fe_{0.15}Ti_{0.85}$	285.0	286.2	289.0
	(56.3)	(22.5)	(21.2)
$Fe_{0.15}Ti_{0.85}$-SCR	285.0	286.0	289.1
	(40.5)	(31.1)	(23.4)
$Cu_{0.10}Ti_{0.90}$	285.0	286.3	288.8
	(60.4)	(25.3)	(14.3)
$Cu_{0.10}Ti_{0.90}$-SCR	285.0	286.2	288.9
	(53.9)	(31.2)	(14.9)

additional component at 291.3 (2.2%) - carbonates

Table S3. Data obtained by performing numerical analysis of high-resolution XPS spectra of O 1s lines. The BE values (eV) and relative areas of components (%) are given.

Sample	Me-O	Ti-O	C-O, O-C=O
$Ce_{0.05}Ti_{0.95}$	528.1<br>(2.2)	529.9<br>(81.9)	531.6<br>(15.9)
$Ce_{0.05}Ti_{0.95}$-SCR	528.1<br>(4.3)	529.9<br>(81.8)	531.3<br>(13.9)
$Ce_{0.10}Ti_{0.90}$	528.1<br>(2.8)	529.9<br>(82.2)	531.6<br>(15.0)
$Ce_{0.10}Ti_{0.90}$-SCR	528.0<br>(2.3)	529.9<br>(81.5)	531.5<br>(16.2)
$Ce_{0.15}Ti_{0.85}$	527.9<br>(1.9)	529.9<br>(88.0)	531.9<br>(10.1)
$Ce_{0.15}Ti_{0.85}$-SCR	528.2<br>(3.4)	529.8<br>(77.8)	531.4<br>(18.8)
$Fe_{0.05}Ti_{0.95}$	527.9<br>(1.8)	529.8<br>(84.0)	531.5<br>(14.2)
$Fe_{0.05}Ti_{0.95}$-SCR	528.2<br>(5.0)	529.7<br>(80.0)	531.3<br>(15.0)
$Fe_{0.10}Ti_{0.90}$	527.9<br>(2.0)	529.9<br>(76.7)	531.5<br>(21.3)
$Fe_{0.10}Ti_{0.90}$-SCR	528.0<br>(2.6)	530.0<br>(82.4)	531.6<br>(15.0)
$Fe_{0.15}Ti_{0.85}$	527.9<br>(2.2)	529.9<br>(81.8)	531.4<br>(16.0)
$Fe_{0.15}Ti_{0.85}$-SCR	528.3<br>(3.9)	530.0<br>(87.4)	532.0<br>(8.7)
$Cu_{0.10}Ti_{0.90}$	528.0<br>(3.6)	529.9<br>(85.1)	531.6<br>(11.3)
$Cu_{0.10}Ti_{0.90}$-SCR	528.1<br>(3.5)	530.0<br>(87.5)	531.6<br>(9.0)

Table S4. Binding energy values (eV) of the Ce 3d lines dominated by multiplet effects. Peaks originating from Ce^{3+} are marked in violet. The proportion of Ce^{3+} species across the spectra is expressed as a percentage.

Sample	v_0	v	v'	v''	v'''	u_0	u	u'	u''	u'''	$Ce^{3+}/(Ce^{3+} + Ce^{4+})$
$Ce_{0.05}Ti_{0.95}$	880.7	882.2	885.7	890.0	899.5	898.3	900.9	904.2	907.5	916.3	66.1
$Ce_{0.05}Ti_{0.95}$-SCR	880.8	882.3	885.8	889.9	900.2	899.1	901.4	904.2	907.8	916.5	67.8
$Ce_{0.10}Ti_{0.90}$	880.8	882.3	885.7	889.8	900.0	898.9	901.0	904.2	907.5	916.6	52.9
$Ce_{0.10}Ti_{0.90}$-SCR	880.7	882.3	885.8	889.7	899.7	898.7	901.0	904.2	907.5	916.6	72.3
$Ce_{0.15}Ti_{0.85}$	880.6	882.4	885.6	889.2	899.0	898.1	901.0	904.0	907.5	916.7	64.3
$Ce_{0.15}Ti_{0.85}$-SCR	880.6	882.5	885.7	889.0	899.2	898.2	901.0	904.0	907.1	916.8	45.9

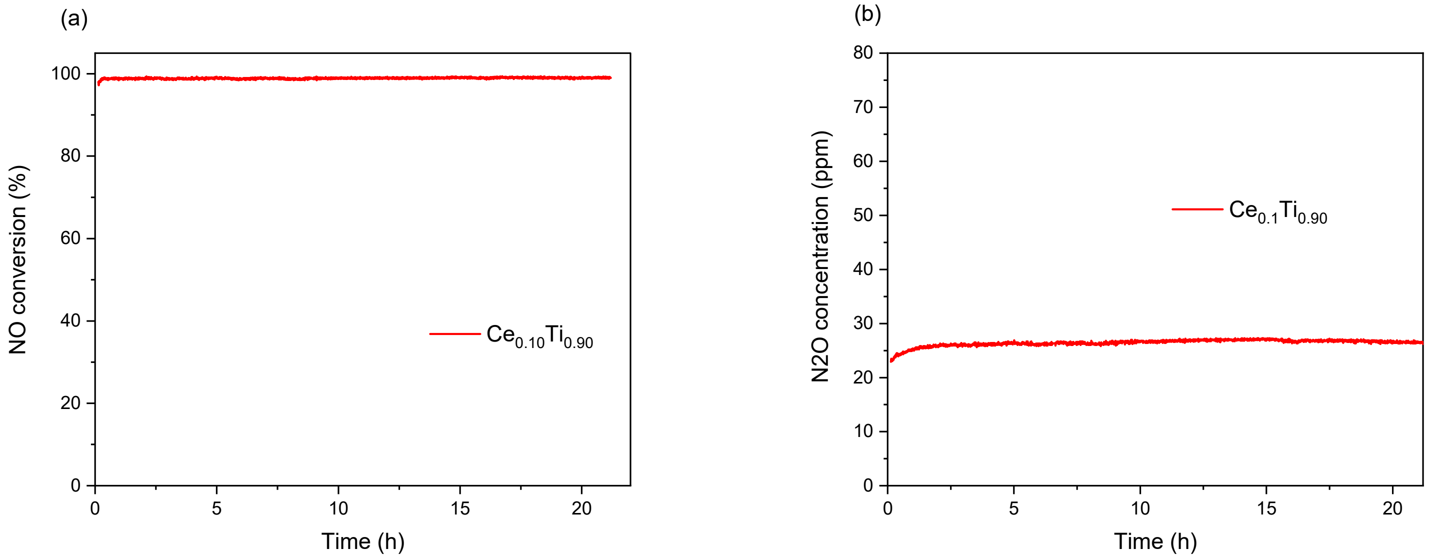

Figure S1. NO conversion (a) and N_2O formation (b) over the $Ce_{0.10}Ti_{0.90}$ catalyst during stability test carried out at 250ºC for 22 hours.

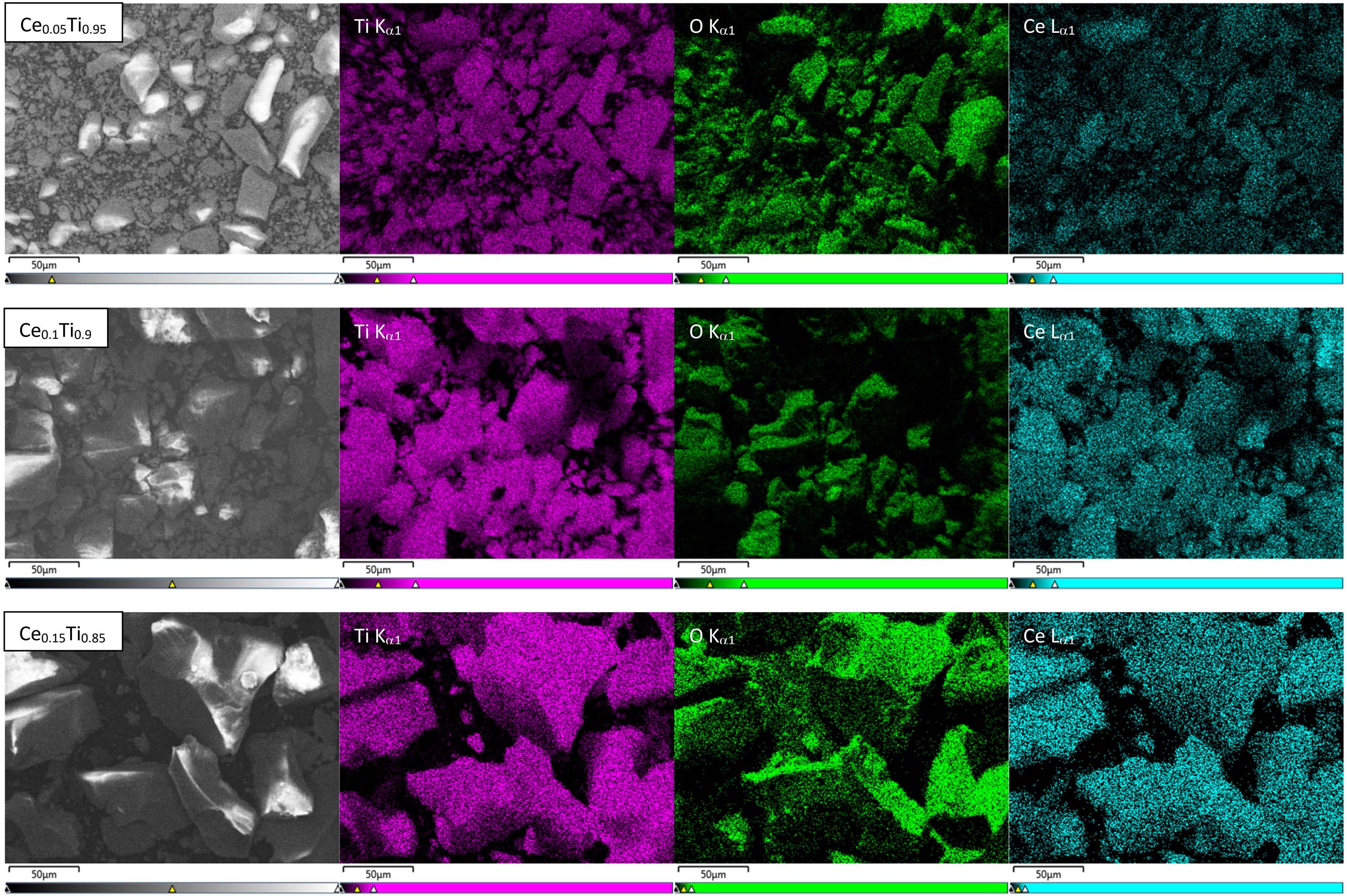

$Ce_{0.05}Ti_{0.95}$
Ti Kα1
O Kα1
Ce Lα1
50μm
$Ce_{0.1}Ti_{0.9}$
Ti Kα1
O Kα1
Ce Lα1
50μm
$Ce_{0.15}Ti_{0.85}$
Ti Kα1
O Kα1
Ce Lα1
50μm

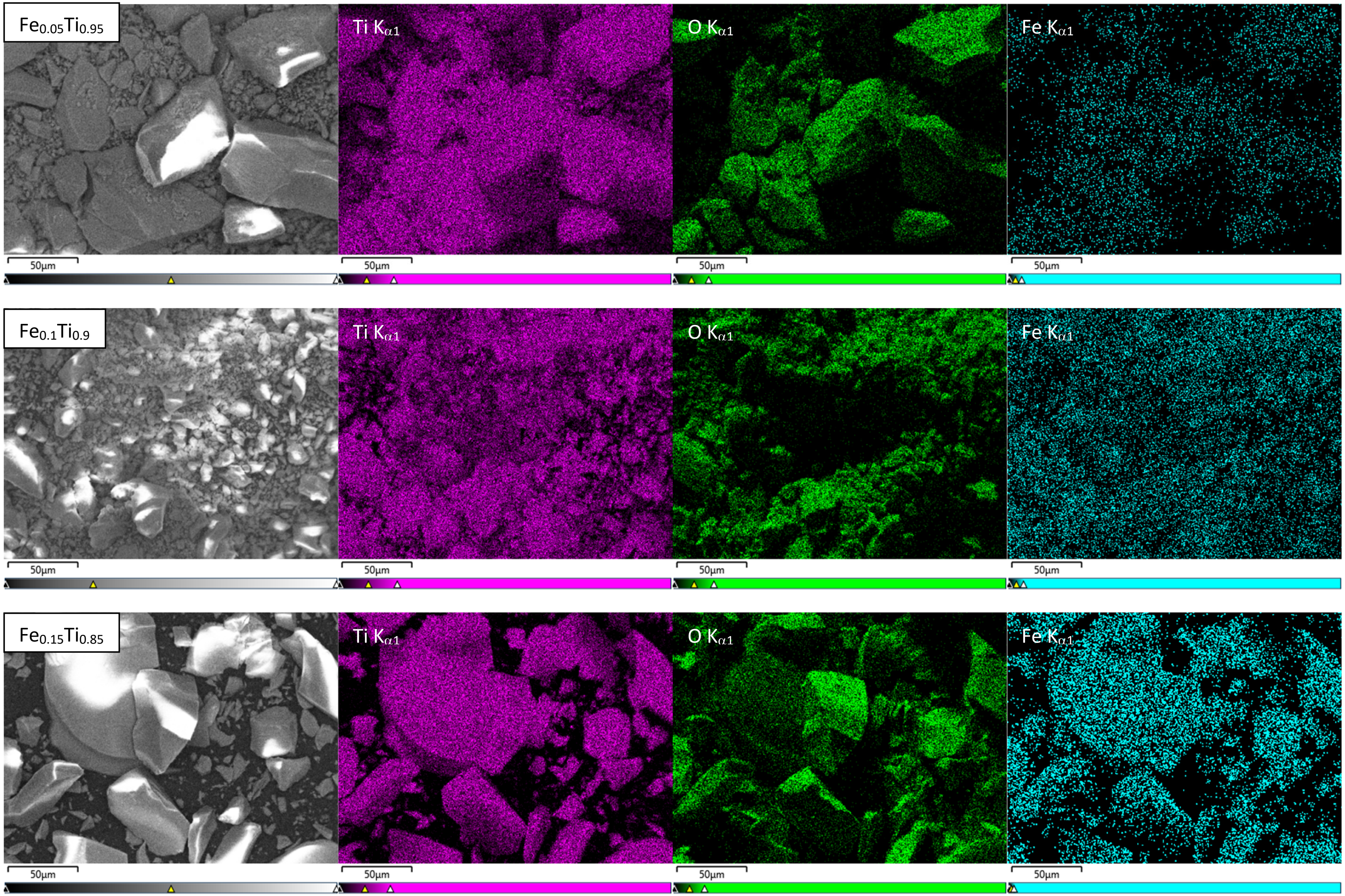

Fe0.05Ti0.95
Ti Kα1
O Kα1
Fe Kα1
Fe0.1Ti0.9
Fe0.15Ti0.85
50µm

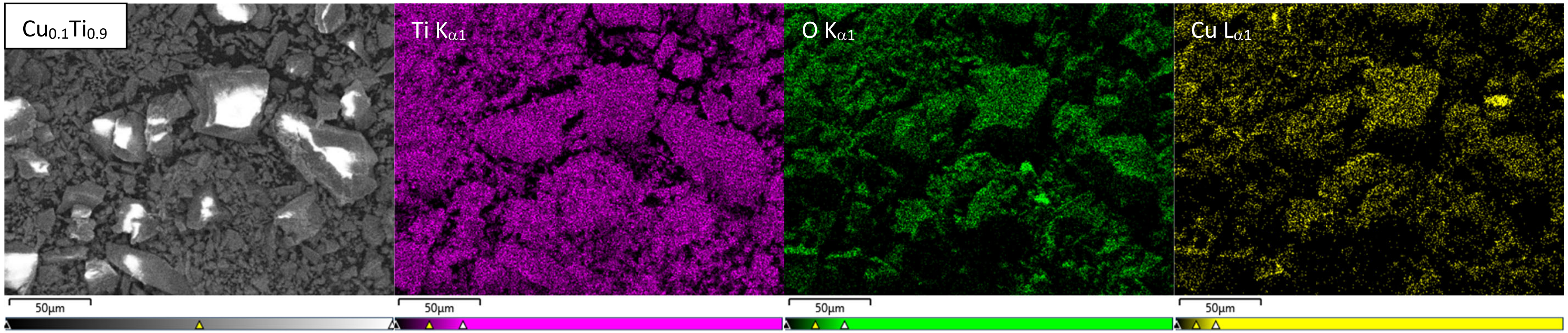

Figure S2. Maps showing the distribution of elements in Ce(Cu, Fe) titania samples obtained using SEM-EDS.
